# Impacts of different vegetation in riparian buffer strips on runoff and sediment loss

**DOI:** 10.1002/hyp.14733

**Published:** 2022-11-01

**Authors:** Robert M. Dunn, Jane M. B. Hawkins, Martin S. A. Blackwell, Yusheng Zhang, Adrian L. Collins

**Affiliations:** ^1^ Net Zero and Resilient Farming Rothamsted Research Okehampton UK

**Keywords:** erosion, flooding, riparian buffers, vegetation management, water quality

## Abstract

Buffer strips continue to feature in the management of agricultural runoff and water pollution in many countries. Existing research has explored their efficacy for reducing environmental problems in different geoclimatic settings but, the evidence on the efficacy of different vegetation treatments is less abundant than that for other buffer strip characteristics, including width, and is more contradictory in nature. With policy targets for various environmental outcomes including water or air quality and net zero pointing to the need for conversion of agricultural land, the need for robust experimental evidence on the relative benefits of different vegetation types in buffer strips is now renewed. Our experiment used a replicated plot scale facility to compare the efficacy of 12 m wide buffer strips for controlling runoff and suspended sediment loss during 15 sampled storms spanning 2017–2020. The buffer strips comprised three vegetation treatments: a deep rooting grass (*Festulolium* cv. Prior), a short rotation coppice willow and native broadleaved woodland trees. Over the duration of the monitoring period, reductions in total runoff, compared with the experimental control, were in the order: willow buffer strips (49%); deciduous woodland buffer strips (46%); grass buffer strips (33%). The corresponding reductions in suspended sediment loss, relative to the experimental control, were ordered: willow buffer strips (44%) > deciduous woodland buffer strips (30%) > grass buffer strips (29%). Given the 3‐year duration of our new dataset, our results should be seen as providing evidence on the impacts during the establishment phase of the treatments.

## 1 INTRODUCTION

Pollution of water by intensive farming continues to be cause for concern for the physicochemical and ecological health of freshwaters (Mateo‐Sagasta et al., 2017). Contaminants moving from agricultural land into freshwater systems include fine‐grained sediment (Pulley & Collins, 2019), purposely applied chemical compounds, for example, fertilizers such as urea (Gilbert et al., 2005), ammonium nitrate (Burt et al., 2011), phosphorus (Haygarth et al., 2005) and other products such as pesticides (Syafrudin et al., 2021). In turn, these emissions not only degrade water quality but also impact detrimentally on freshwater ecology across all trophic levels (Collins et al., 2011; Jones et al., 2014; Jones, Collins, et al., 2012; Jones, Murphy, et al., 2012; Kemp et al., 2011).

Buffer strips have been utilized as a means of reducing the movement of pollutants from agricultural land into watercourses for many years (Barling & Moore, 1994; Hickey & Doran, 2004). The form of the vegetation may take that of a grass verge at the edge of the field where no targeted planting of chosen species is undertaken and natural colonization is allowed to determine the dominant form of vegetation. Alternatively, targeted planting of specific grasses and woody plants has been utilized to vegetate buffer strips, with consequent effects on landscape aesthetics, biodiversity and interactions with the local watercourse and its ecology (Cole et al., 2020). Choices of the type of plants that can be deliberately planted within a buffer strip range from herbaceous grasses and forbs to small woody shrubs with multiple stems to taller woody tree species. The physiognomy of the plants may affect the runoff, the movement of pollutants including fine‐grained sediment, or both (Roberts et al., 2012). The interaction‐potential the buffer strip has for removing pollutants from the runoff leaving the field from upslope may thus change depending on the form of planting used to vegetate the buffer strip. Here, the form of planting chosen may affect the ability of the buffer strip to remove a priority pollutant within a local area, and as a result, some degree of potential exists to optimize buffer vegetation to ameliorate specific local concerns over particular pollutants or, alternatively, to address multiple issues (Stutter et al., 2012).

Water pollution and flooding events associated with the movement of agricultural run‐off have been reduced due to the interaction of water and vegetation within buffer strips. However, the ability of a buffer strip to provide such services continuously may be reduced or lost over time if the buffer strip becomes saturated with fine‐grained sediment or nutrients (Valkama et al., 2018). To alleviate the potential for saturation of nutrients, planned removal of buffer strip vegetation can be implemented. For grass buffer strips, mowing and/or grazing can reduce the standing crop within the strip and cause compaction by trafficking or trampling. Access to strips may negate the possibility of using machinery in some circumstances (e.g., steeply sloped land), and refusal by grazing animals to consume standing vegetation may affect the amount of vegetation removed. The age of a grass dominated buffer strip may need to be considered if grazing animals are the only option available to reduce the standing crop. Woody plants can be harvested for their timber within a planned management system, and act as a means of both removing nutrients from the strip as well as reinvigorating plant growth rates, and thus facilitating the further removal of nutrients entering the buffer strip.

In England, implementation of water pollution interventions on farms, including buffer strips, is driven by a combination of regulation, incentivization in the form of agri‐environment schemes and the delivery of on‐farm advice for win‐wins. Here, improved uptake rates by farms can be encouraged by robust scientific evidence on the efficacy of buffer strips for controlling runoff and pollution losses. Existing work examining the efficacy of buffer strips for environmental good has focussed on both external and internal factors (Eck, 2000). The former encompass the phase (i.e., particulate, dissolved) and delivery pathway (i.e., surface, subsurface) of the incoming pollution, whereas the latter include buffer width and vegetation cover. Advice delivery has tended to focus on buffer width in the case of internal controls since this is the easiest component of management to influence via farm management and existing evidence on varying efficacy for runoff and water pollution reduction, including width, can be readily extracted from a number of comprehensive reviews (e.g., Barling & Moore, 1994; Collins et al., 2009; Dorioz et al., 2006; Hickey & Doran, 2004; Kay et al., 2009). Beyond buffer strip width, the existing evidence on the effects of different vegetation cover remains less easy to generalize. Some work suggests that for the same buffer strip width, different vegetation cover impacts efficacy for pollution control by at most 20% (Dorioz et al., 2006). Other studies report very limited or no effect of vegetation cover (e.g., Schmitt et al., 1999; Uusi‐Kämppä et al., 2000). In other cases, the results of investigations comparing herbaceous and woody vegetation in buffer strips report both a lack of (Daniels & Gilliam, 1996; Syversen, 1995) and detectable (Cooper et al., 1986; Parsons et al., 1994) differences in pollution reduction, with the latter suggesting better performance by herbaceous cover.

Given the above context, the new study detailed herein was undertaken to assess the impact of three different vegetated buffer strips on runoff and sediment loss to contribute to the evidence base. The research project was planned to provide replicated evidence on buffer strip efficacy and to engage multiple stakeholders with this evidence given the ongoing inclusion of buffer strips in agricultural policy in the United Kingdom and beyond. This paper reports the preliminary results for the efficacy for reductions in runoff and sediment loss using our new dataset.

## 2 METHODOLOGY

### 2.1 Study site description

The assessment of buffer strip efficacy was undertaken on experimental plots located at the Rothamsted Research North Wyke site in Devon, UK (50°46′31.3″N 3°55′41.6″W). This site has a mean annual rainfall of 1032 mm y^−1^, a mean maximum temperature of 13.5°C and mean minimum temperature of 6.7°C (1982–2019; see Table 1 for mean monthly values).

**TABLE 1:** Observed long‐term climate data for the study site for 1982–2019 (±standard error of the mean).

	January	February	March	April	May	June	July	August	September	October	November	December
Mean total monthly rainfall (mm)	120 ± 9	88 ± 10	81 ± 6	65 ± 6	65 ± 6	59 ± 6	57 ± 6	66 ± 6	71 ± 6	113 ± 8	117 ± 9	129 ± 10
Mean monthly maximum temperature (°C)	7.8 ± 0.3	7.9 ± 0.3	9.9 ± 0.2	12.3 ± 0.3	15.3 ± 0.2	18.1 ± 0.2	20.0 ± 0.3	19.7 ± 0.3	17.5 ± 0.2	14.2 ± 0.2	10.7 ± 0.2	8.5 ± 0.3
Mean monthly minimum temperature (°C)	2.6 ± 0.3	2.2 ± 0.3	3.4 ± 0.2	4.4 ± 0.2	7.1 ± 0.2	9.9 ± 0.1	12.0 ± 0.1	12.0 ± 0.1	10.1 ± 0.2	8.0 ± 0.3	5.3 ± 0.2	3.5 ± 0.3

*Source*: Crown copyright Met Office, UK.

The site is situated upon a bedrock of clay bearing shales of the Carboniferous Crackington Formation, with overlying soils represented by a poorly drained Hallsworth series pelo‐stagnogley soil (FAO classified as Stagni‐Vertic Cambisol; Harrod & Hogan, 2008). The stony clay loam topsoil comprises 16%, 48% and 36% of sand, silt and clay, respectively. Below the topsoil layer (~0 to 30 cm), the subsoil (~30 to 160 cm) is impermeable to water and is seasonally waterlogged; most excess water moves by surface and sub‐surface lateral flow across the clay layer (Orr et al., 2016), with the experimental area having a slope of 8°.

### 2.2 Experimental set‐up

**FIGURE 1:**
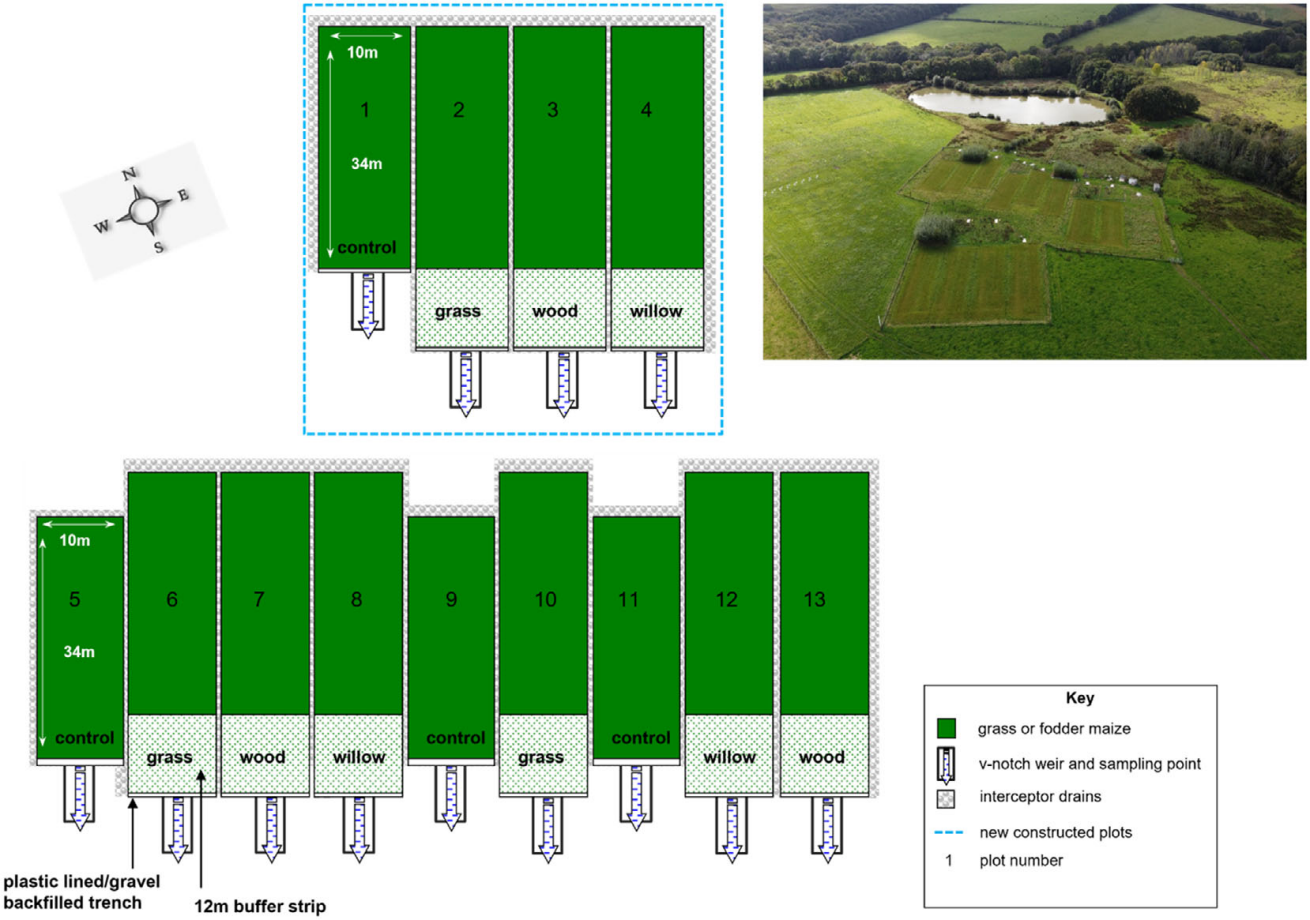
Schematic of the experimental platform and aerial photograph of the experimental facility, showing the replicated buffer plots.

A total of 12 plots (Figure 1) were used in the present study, eight of which were created for a previous buffer strip experiment established in 2008 (Final Report BBSRC SARIC project BB/N004248/1) and four of which were newly created in the same location for the work described herein. Each plot was hydrologically‐isolated from the neighbouring plots via the installation of gravel‐filled French drains on the upper and side edges with waterproof membrane on the bottom and side of the drain adjacent to the plot. The plots measured 34 m × 10 m, with nine of the plots having an extra 12 m × 10 m area which constituted the buffer strip (i.e., a total area of 46 m × 10 m). The three plots lacking the extra buffer strip areas were treated as the experimental controls. Three replicate plots were set up as buffer strips for each of three different types of vegetation cover which was established in 2016 in the uppermost 10 m of the buffer section. The remaining bottom 2 m of the buffer section of each replicated plot with a buffer treatment was left as an uncut grass strip (to replicate the minimum requirements of farmers for buffer strips in agricultural policy for England at the time the experiment was initiated). The three types of buffer strip vegetation consisted of a deep rooting grass (*Festulolium* cv. Prior), short rotation coppice willow and native broadleaved woodland trees. Prior to sowing the *Festulolium* grass seed, the existing grassland sward was removed using glyphosate herbicide and the ground rotovated. An initial sowing in October 2017 failed to establish sufficient *Festulolium*, and a second sowing was undertaken in September 2018. The willow treatment comprised 200 stems in the 10 m × 10 m buffer area (i.e., equivalent to 20 000 stems ha^−1^) equally split between five cultivars (Endurance, Terra Nova, Cheviot, Hambleton and Mourne). The area was pre‐treated with glyphosate herbicide to remove the existing grassland sward prior to planting willow, with stems of 30 cm length inserted flush to ground level in May 2017. The layout of the planting consisted of five pairs of lines 0.75 m apart, with a gap of 1.5 m between pairs of rows. Willow stems were randomly inserted into the ground at 0.5 m spacing along the rows, which ran perpendicular to the slope of the field. Four‐strand electric fencing was erected around the outer edge of each buffer strip with willow to provide protection from browsing deer. The native broadleaf tree treatment consisted of six species – hornbeam (*Carpinus betulus* L.), sweet chestnut (*Castanea sativa* Mill.), hazel (*Corylus avellana* L.), pedunculate oak (*Quercus robur* L.), small‐leaved lime (*Tilia cordata* Mill.) and wych elm (*Ulmus glabra* Huds.). Five bare‐root trees of each species were planted (i.e., equivalent to 3000) in each 10 m × 10 m buffer strip area in December 2017. Five rows running perpendicular to the slope of the field, each with six trees, were randomly planted 1.75 m apart, with each row offset by approximately 0.85 m to the neighbouring row. Prior to planting, the existing grassland sward was removed using a glyphosate herbicide spray. The trees each had a 1.2 m tall green plastic tree guard held in place via a 1 m wooden stake, with a four‐strand electric fence surrounding the outer edge of the buffer strip to protect the trees from browsing deer.

The upper part of each of the 12 replicate plots was treated as an agriculturally managed area, with a grass silage crop being cut twice in 2017, three times in 2018, and once in 2019. In April 2019, the existing sward in this agriculturally managed area was removed using glyphosate herbicide, ploughed, rotovated and a maize crop (Garni CS cultivar with methiocarb and fludioxonil coatings) established to test buffer performance with a higher risk crop upslope. A post emergence herbicide was applied 6 weeks after drilling (Nico Pro 4SC [Nicosulfuron] at 1 L/ha). Details of fertilizer applications on the agriculturally managed areas are given in Table 2.

**TABLE 2:** Activity dates and rates of fertilizer applications on the agricultural plots upslope of the buffer strip treatments.

Grass silage date	Nitrogen (as NH_4_NO_3_) (kg ha^−1^)	Phosphorus (as TSP) (kg ha^−1^)	Potassium (as M of P) (kg ha^−1^)	Lime (kg ha^−1^)
May‐17	231	152	133	250
July‐17	144	54	166	
September‐17			50	
*sum*	375	206	349	
Apr‐18	231	217	133	
May‐18	145	54	167	
Jul‐18	145	33	133	
Sep‐18			50	
*sum*	521	304	483	

Maize date	Nitrogen (kg ha^−1^)	Slurry phosphorus (kg ha^−1^)	Potassium (kg ha^−1^)
May‐19	60	28	80
	Nitrogen (as NH_4_NO_3_)	Phosphorus (as TSP)	Potassium (as MofP)
May‐19	202	159	265
*sum*	262	187	345

Abbreviations: M of P, muriate of potash; TSP, triple super phosphate.

### 2.3 Experimental instrumentation

Runoff from each of the 12 experimental plots passed into a gravel‐filled trench located at the bottom edge of each plot. An impermeable membrane was installed at a depth of approximately 0.5 m below the ground on the bottom and opposite side of the trench to the plot to capture both surface and sub‐surface lateral flowing water exiting the experimental plots. A V‐shape base of the trench ensured that all water exited the trench at the same point, where it was channelled into a V‐notch weir fitted with a baffle plate to smooth out changes in the height of the water. The height of the water within the weir was monitored via an ADCON LEV1 level sensor (0–1 m range) positioned within a stilling well. Data were recorded every minute via a Delta T GP1 data logger as millivolt values and converted to discharge using a rating equation established under laboratory calibration conditions. To allow for the areal difference between the control plots (340 m^2^) and the buffer treatment plots (340 m^2^ + 120 m^2^ = 460 m^2^), the runoff volumes were adjusted by multiplying them by a correction factor calculated using the formula:

(1) $$\text{Correction factor}=1-\frac{\text{Buffer area}}{\text{Total plot area}}$$

A total of 15 storm runoff events were sampled between 2017 and 2020. In March 2020, data collection ceased due to the Covid‐19 pandemic. On that basis, our results are reported for individual monitoring years spanning April–March, rather than the more conventional water year (October–September) for the northern hemisphere. This reporting period also aligns better with the farm management year for grassland systems wherein, fertilizers for encouraging grass growth are applied in spring and fodder maize is also sown.

Water samples were collected using an Envitech SampSys autosampler. A total of 24 samples could be collected from each replicate plot, with millivolt (mV) readings from the level sensor used to trigger the initiation of sampling on a 10 mV staging basis. Depending on the rainfall event and when taken on the hydrograph, the number of samples selected for analysis ranged from 6 to 12, with an overall average across the study period of 10 per event. Sampling periods were established via Meteorological Office weather forecasts, with a delayed start via the internal clock of the SampSys used to gain samples from both the rising and falling limbs of the hydrograph of any individual sampled storm runoff event.

### 2.4 Water sample processing and analysis

Individual water samples from each sampled storm event were selected to represent the full extent of the storm hydrograph and analysed for suspended sediment concentration. Water samples were refrigerated at 5°C following collection until analysis. Suspended sediment content was assessed by filtering a sub‐sample of water of known volume through Whatman GF/C filter paper (Whatman, Buckinghamshire, UK) with a particle retention size of 1.2 μm, drying at 105°C and weighing to assess the mass gained (UK Standing Committee of Analysts, 1980 #597). Suspended sediment loads were calculated by multiplying discharge volume (L) by the concentration of suspended sediment (mg L^−1^) for each sample point. Total loads were calculated using trapezoidal integration of the timeseries curve.

## 3 RESULTS

### 3.1 Rainfall event information

Information for the individual rainfall events that were sampled is provided in Table 3. The largest rainfall event (28.1 mm) occurred on 14 March 2018 (event number 7) with a duration of 2340 min following a previous 5‐day rainfall total of 26.5 mm. The smallest rainfall event (7.1 mm) sampled was on 10 December 2019 (event number 15) with a duration of 2160 min following a previous 5‐day rainfall total of 24.6 mm. The longest duration of rainfall for a sampled event was 4080 min between 23 January 2018 and 25 January 2018 (event number 6) with a rainfall total of 17.4 mm and a maximum hourly rainfall of 9.9 mm following a previous 5‐day rainfall total of 23.5 mm. This latter sampling formed part of a named storm event ‐ Storm Georgina (https://www.metoffice.gov.uk/about-us/press-office/news/weather-and-climate/2018/storm-georgina-brings-strong-winds-to-the-uk-and-ireland) and the impact of this event on suspended sediment loss is described later. All the rainfall events sampled were preceded by rainfall within the previous 5 days, ranging from a maximum of 50.2 mm (event number 8) to a minimum of 2.1 mm (event number 14).

**TABLE 3:** Rainfall characteristics of the sampled events.

Event number	Date	Total rainfall (mm)	Maximum hourly rainfall (mm)	Duration (min)	P5^a^ (mm)
1	22 March 2017	15.5	4.0	1680	16.4
2	3 January 2018	17.4	2.7	1740	45.2
3	4 January 2018	9.9	2.9	1200	41.4
4	18 January 2018	13.0	2.9	1380	26.5
5	22 January 2018	8.2	1.3	1680	26.0
6	24 January 2018	17.4	9.9	4080	23.5
7	15 March 2018	28.1	3.2	2340	26.5
8	12 November 2018	26.7	3.6	2400	50.2
9	11 February 2019	10.3	3.8	1380	35.5
10	06 March 2019	20.0	3.4	2700	32.1
11	13 March 2019	16.6	5.9	1920	14.9
12	12 June 2019	13.0	3.8	1980	48.5
13	25 October 2019	21.4	1.9	2400	25.2
14	25 November 2019	18.9	2.7	2640	2.1
15	11 December 2019	7.1	2.7	2160	24.6

^a^ 5‐day antecedent total rainfall.

### 3.2 Reductions in runoff for the buffer strips with different vegetation

**FIGURE 2:**
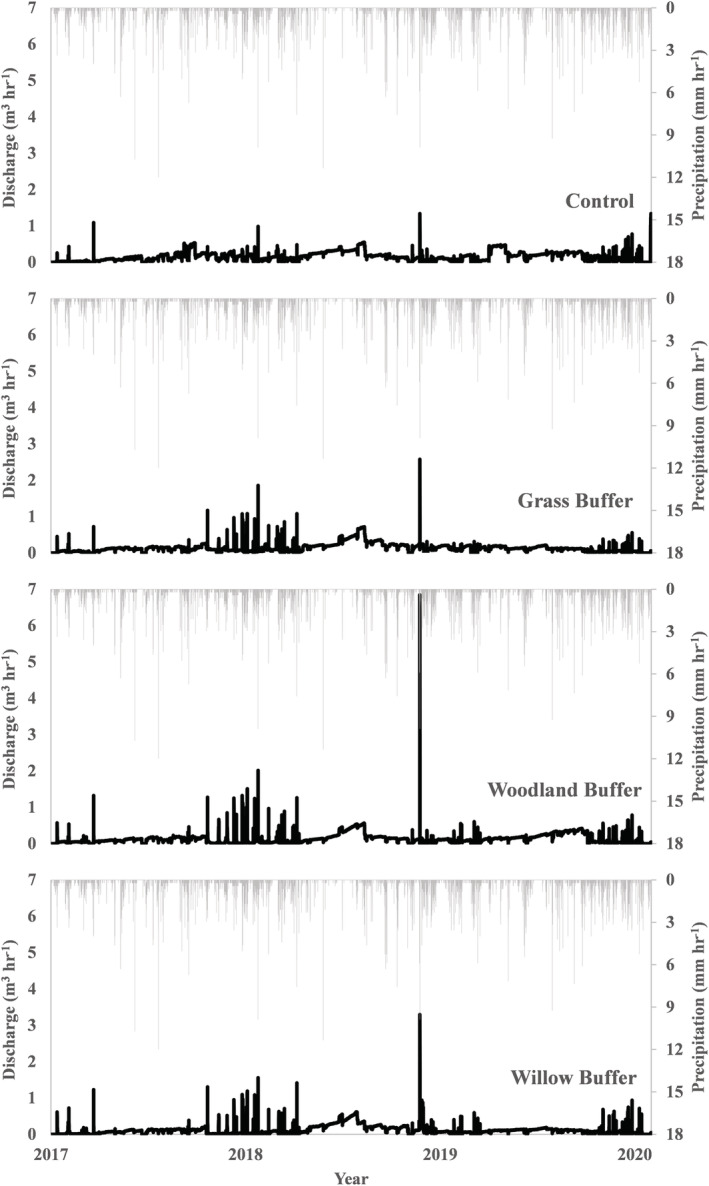
Mean discharge from each buffer strip treatment (black line) and hourly precipitation (grey bars) for the entire monitoring period (2017–2020).

Discharge data (m^3^) for all treatments spanning the entire monitoring period (2017–2020) are presented graphically as a timeseries in Figure 2 and summarized on an annual basis in Table 4.

**TABLE 4:** Mean total adjusted discharge volumes (m^3^) for each treatment and year.

Buffer strip treatment	April 2017–March 2018	April 2018–March 2019	April 2019–March 2020
Control	924	1196	1120
Grass buffer	635 (859)	1135 (1536)	401 (543)
Willow buffer	398 (539)	891 (1206)	372 (503)
Woodland buffer	531 (718)	698 (944)	534 (723)

*Note*: Unadjusted discharge volumes in parentheses.

The percentage changes in discharge for the each buffer strip treatment for each rainfall event, compared with the control, are given in Table 5 and were calculated using the formula:

(2) $$\frac{x_{c}-x_{t}}{x_{c}}\times 100$$

Where *x*_c_ is the control treatment and *x*_t_ is one of the other buffer strip treatments. Positive values represent a reduction in volume of discharge compared with the control whereas negative values represent an increased volume of discharge; that is, no reduction. During the first year of the establishment of the buffers, except for the grass buffer in event number 1, there was no reduction in discharge for the first 7 events that were sampled. Event number 8 (12 November 2018) showed a reduction in discharge from all buffer treatments, compared with the control, in the order of willow > grass > woodland. It is not clear as to why there was a marked change in performance of all the buffer treatments other than the total rainfall for this event was 26.7 mm following a 5‐day antecedent period (P5) of rainfall totalling 50.2 mm (Table 3); the largest of all the sampled events. This pattern was repeated in event 12 (12 June 2019) with a rainfall total of 13.0 mm following a P5 total of 48.5 mm, the second highest 5‐day antecedent rainfall of all the sampled events and where reductions again followed the order: willow > grass > woodland. It was not until sampled event numbers 14 and 15, during the latter months of 2019, that all the buffer treatments showed a reduction in discharge compared with the control, though the order of efficacy was different for each event and did not follow the pattern observed previously in events 8 and 12.

**TABLE 5:** Percentage (%) change in discharge volumes for each buffer strip treatment relative to the control for each rainfall event.

Event number	Date	Grass buffer	Woodland buffer	Willow buffer
1	22 March 2017	48	−7	0
2	3 January 2018	−159	−245	−114
3	4 January 2018	−109	−201	−120
4	18 January 2018	−114	−194	−147
5	22 January 2018	−93	−235	−54
6	24 January 2018	−63	−87	−27
7	15 March 2018	−52	−65	−17
8	12 November 2018	53	49	66
9	11 February 2019	−23	−183	−130
10	6 March 2019	21	−107	−86
11	13 March 2019	−42	−43	−10
12	12 June 2019	31	26	62
13	25 October 2019	−5	−201	43
14	25 November 2019	45	4	17
15	11 December 2019	1	26	33

In the case of the grass buffer strip treatment, the reduction in discharge (Table 6) compared with the control, ranged from 5% (2018–2019) to 64% (2019–2020). The corresponding efficacy for the willow buffer strip treatment ranged from 25% (2018–2019) to 67% (2019–2020). For the deciduous woodland buffer strip treatment, the reductions in discharge, compared with the control, ranged from 42% (2018–2019) to 52% (2019–2020). For the entire monitoring period (2017–2020; Table 6), reductions in discharge were in the descending order: willow buffer strips (49%) > deciduous woodland buffer strips (46%) > grass buffer strips (33%). A hydraulic conductivity study undertaken during 2019 showed that all the buffer strip treatments had a significantly higher field saturated hydraulic conductivity (Kfs; mm h^−1^) rate than the upslope cropped areas (*p <* 0.01). In addition, the woodland and willow buffers had a significantly higher (*p <* 0.01) Kfs rate than the grass buffers (data not shown). Clearly, the relative impacts of buffer strip treatments show higher event‐based variations. However, there is indication that there are more positive changes with the progression of buffer maturity as shown in later years (Table 5), especially in the case of the woodland and willow buffer strip treatments.

**TABLE 6:** Relative change (%) in annual and total discharge volumes for each buffer strip treatment relative to the control.

Buffer strip treatment	April 2017–March 2018	April 2018–March 2019	April 2019–March 2020	Total
Grass buffer	31	5	64	33
Willow buffer	57	25	67	49
Woodland buffer	43	42	52	46

#### 3.2.1 Discharge:rainfall ratios

The discharge:rainfall ratios for each rainfall event sampled are given in Table 7 and show that in the first year following their establishment, discharge in the buffer strip treatments occasionally exceeded rainfall and generated more flow than the control (event numbers 3, 5), though the reason for these results is unclear. These early data are most probably artefacts associated with the settling down of the experimental set‐up including the instrumentation, and possibly also due to soil disturbance in the process of setting up the buffer treatments. From event number 5 onwards, ratios varied between 0.11 and 0.73 with an overall average value of 0.33, though statistical analysis showed that any differences between the treatments were not statistically significant (data not shown). The average value is slightly lower than the reported annual standard percentage runoff (SPR) associated with the on‐site soil series, which is 40% (Boorman et al., 1995).

**TABLE 7:** Discharge:rainfall ratios by treatment for each sampled rainfall event.

Event number	Control	Grass buffer	Woodland buffer	Willow buffer
1	0.56	0.34	0.53	0.94
2	0.29	0.72	0.73	0.95
3	0.49	1.19	1.34	1.72
4	0.23	0.51	0.52	0.74
5	0.34	0.70	0.70	1.02
6	0.35	0.68	0.59	0.73
7	0.25	0.45	0.35	0.47
8	0.39	0.25	0.15	0.21
9	0.24	0.27	0.61	0.70
10	0.19	0.21	0.40	0.54
11	0.12	0.21	0.21	0.21
12	0.17	0.20	0.14	0.13
13	0.16	0.18	0.11	0.33
14	0.25	0.32	0.35	0.40
15	0.55	0.54	0.32	0.25

#### 3.2.2 Time to peak discharge compared with time to peak rainfall

Analysis of the data for time to peak discharge compared with that of peak rainfall for each of the sampled events showed that differences between the treatments were not statistically significant (data not shown). This is attributed to the short length of the flow paths being monitored. While time to peak discharge can be important for flood mitigation, the current plot set‐up was not designed to examine this specific effect.

### 3.3 Reductions in suspended sediment loss for the buffer strips with different vegetation

**FIGURE 3:**
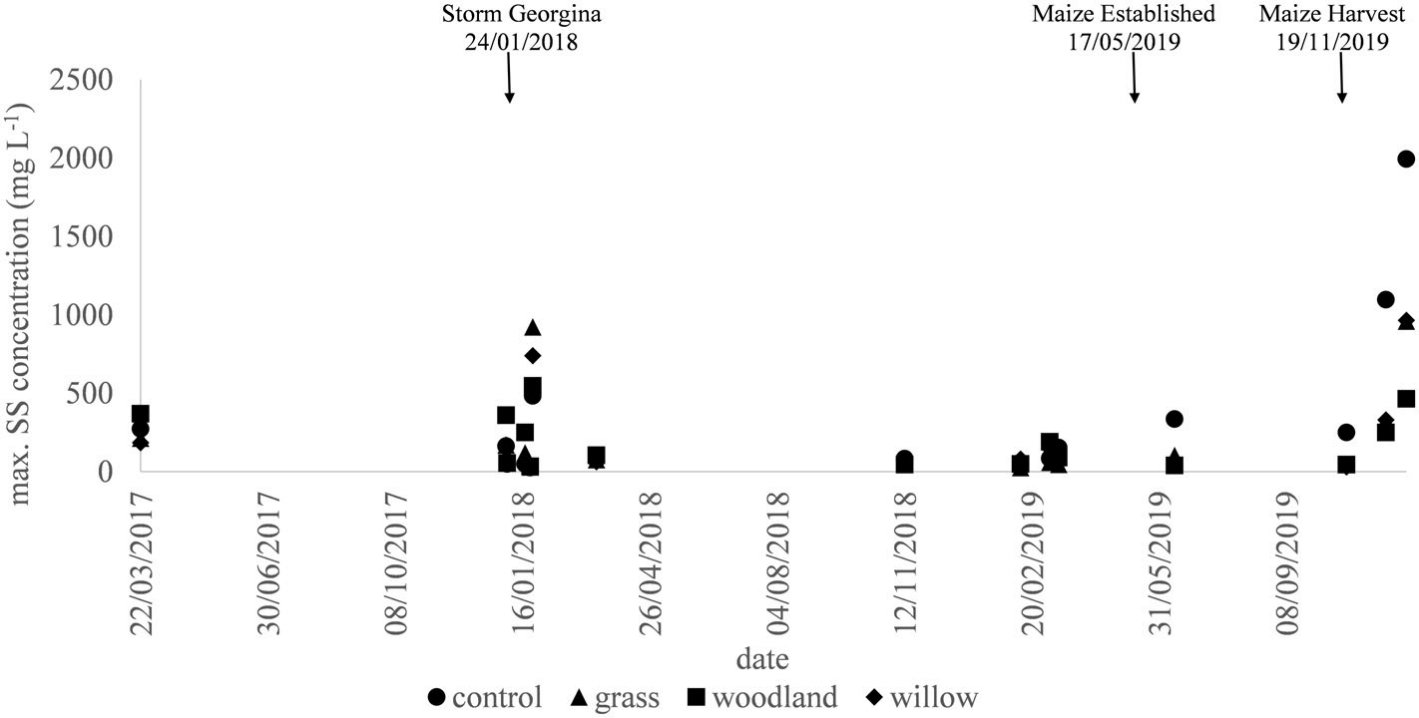
Maximum suspended sediment (SS) concentration (mg L^−1^) for each rainfall event for the control (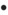), grass buffers (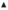), woodland buffers (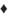) and willow buffers (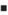).

The maximum concentration of suspended sediment (mg L^−1^) for each event is shown in Figure 3, alongside key land management changes over the study period. Most noticeable, are the maximum concentrations during the rainfall event sampled on 24 January 2018 that coincided with Storm Georgina where concentrations were up to 900 mg L^−1^ from the grass buffer strip compared with the willow buffer (740 mg L^−1^), woodland buffer (546 mg L^−1^) and control (483 mg L^−1^). The higher concentration from the grass buffer treatment is most likely attributable to reseeding operations for establishment of deep rooting grass during the preceding autumn. Figure 3 also shows that following the maize harvest on 19 November 2019, maximum suspended sediment concentrations in the ensuing rainfall events reached up to 2000 mg L^−1^ from the control compared with the willow buffer (965 mg L^−1^), grass buffer (960 mg L^−1^) and woodland buffer (465 mg L^−1^). These results suggest that the buffer positive impact son reducing the volume of discharge is also reflected in the associated suspended sediment concentrations. This temporal dynamic demonstrates the interaction of extreme weather and risky land management activities. The setup of the buffer strip experiment disturbed soil structure, removed near surface litter and reduced ground cover. Clearly, the timing of such activities can have significant impacts on the initial efficacies of the buffer strip treatments.

Table 8 presents the estimates of suspended sediment loss for each individual year and the entire monitoring period. Annual losses from the control treatment ranged between 8.5 kg ha^−1^ (±10.1 kg ha^−1^) in 2018–2019 and 111.8 (±154.9 kg ha^−1^) in 2019–2020. In comparison, the corresponding losses from the replicate plots with grass buffer strips were 3.3 kg ha^−1^ (±4.2 kg ha^−1^) in 2018–2019 and 40.9 kg ha^−1^ (±21.4 kg ha^−1^) in 2017–2018 and from those with willow buffer strips 6.4 kg ha^−1^ (±6.1 kg ha^−1^) in 2018–2019 and 42.8 kg ha^−1^ (±31.2 kg ha^−1^) in 2019–2020. Sediment losses from those plots served by deciduous woodland buffer strips ranged between 6.8 kg ha^−1^ (±6.1 kg ha^−1^) in 2018–2019 and 26.6 kg ha^−1^ (±6.8 kg ha^−1^) in 2017–2018.

**TABLE 8:** Annual and total losses (kg ha^−1^ equivalent) of suspended sediment (standard deviations in parentheses).

Buffer strip treatment	April 2017–March 2018	April 2018–March 2019	April 2019–March 2020	Total
Control	22.2 (±8.2)	8.5 (±10.1)	111.8 (±154.9)	142.5
Grass buffer	40.9 (±21.4)	3.3 (±4.2)	11.9 (±14.8)	56.1
Willow buffer	31.6 (±9.6)	6.4 (±6.1)	42.8 (±31.2)	80.7
Woodland buffer	26.6 (±6.8)	6.8 (±6.1)	20.6 (±11.8)	53.9

The efficacy for sediment load reduction for each sampled event (Table 9) followed a similar pattern as was observed with the discharge data in that there was high event‐based variation but an indication of an improvement in efficacy with buffer maturity, especially in the case of the woody treatments.

**TABLE 9:** Percentage (%) change in suspended sediment load for each buffer strip treatment relative to the control for each rainfall event.

Event number	Date	Grass buffer	Woodland buffer	Willow buffer
1	22 March 2017	32	−65	68
2	3 January 2018	−341	−280	−177
3	4 January 2018	−218	−62	−124
4	18 January 2018	−456	−209	−78
5	22 January 2018	−156	−381	−76
6	24 January 2018	−216	−96	−164
7	15 March 2018	−202	−94	−29
8	12 November 2018	60	50	53
9	11 February 2019	17	−81	−112
10	6 March 2019	−42	−588	−163
11	13 March 2019	29	−81	−72
12	12 June 2019	−83	−64	40
13	25 October 2019	47	33	79
14	25 November 2019	95	88	86
15	11 December 2019	88	98	86

Relative to the experimental control, suspended sediment loss (Table 10) was reduced by the grass buffer strips by between 0% (2017–2018) and 94% (2019–2020), compared with 0% (2017–2018) to 89% (2019–2020) for the willow buffer strips and 0% (2017–2018) to 76% (2019–2020) for the woodland buffer strips. For the entire monitoring period (2017–2020), reductions in total monitored suspended sediment loss were in the descending order: willow buffer strips (44%) > deciduous woodland buffer strips (30%) > grass buffer strips (29%).

**TABLE 10:** Percentage (%) reductions (per hectare) in annual and total suspended sediment loss for each buffer strip treatment relative to the control.

Buffer strip treatment	April 2017–March 2018	April 2018–March 2019	April 2019–March 2020	Total
Grass buffer	−218	41	94	29
Willow buffer	−109	−129	89	44
Woodland buffer	−139	−45	76	30

*Note*: Negative numbers indicate an increase in losses, whilst positive numbers indicate a decrease in losses.

## 4 DISCUSSION

Overall, our results suggest that reductions in discharge in the individual sampled events (Table 5) indicate that the buffer treatments are reaching a stage of maturity that has begun to have a positive impact on hydrology. Continuation of the study over more years may have been able to confirm this. The storm period results suggest that the performance of the types of vegetated buffers tested in this study may be influenced by the amount of rainfall combined with antecedent soil moisture conditions. There was no clear treatment effect on time to peak discharge which is surprising given that hydraulic conductivity rates were greater under the woody buffers.

The results for the establishment phase of the buffer treatments therefore suggest that woody treatments improve sediment trapping the most. Clearly, these preliminary results might change as the vegetation treatments mature over time. The zero efficacy in the first monitoring year across all buffer treatments and in the second monitoring year for the willow and deciduous woodland buffers reflected the soil disturbance associated with the establishment of the three vegetation treatments. The impact of soil disturbance associated with the installation of other sediment mitigation measures on farms, such as channel bank reprofiling and fencing, has been reported by other studies (Lloyd et al., 2016). For the establishment period monitored in this study, our results suggest that the grass buffer strip treatment matures faster with respect to sediment trapping than the other two woody vegetation treatments. Over time, the potential for buffer strip saturation with trapped fine‐grained sediment could be expected to increase.

Direct comparisons of experimental results for buffer strip efficacy are typically compromised by various factors. These include the contrasting climate, soil types, runoff lengths, vegetation types and agricultural practices of research sites. Additional potentially confounding factors include deployment of different research infrastructure and study scales and durations. Nonetheless, it is useful to interpret our new data on buffer strip efficacy for reducing sediment loss in the context of existing evidence. Working on 6 m buffer strips with fescue, shrubs and trees, serving 3% slopes, Borin et al. (2005) reported a sediment trapping efficacy of 93%. Schmitt et al. (1999), comparing 7.5 and 15 m grass, shrub and sorghum buffers, serving a slope of 6.7%, reported a sediment trapping efficacy range of 63%–93%. Syversen (1995), testing 3, 10 and 15 m grass and shrub buffer strips serving slopes of 14% and 28%, reported efficacies of 61%–91%. Schwer and Clausen (1989) working on 26 m wide grass buffer strips, serving slopes of 2%, reported a sediment trapping efficacy of 95%. Across the existing scientific literature reporting reductions in sediment loads due to buffer strips, the efficacy range is typically 40%–100% with reductions of >50% commonplace (Dorioz et al., 2006). Given the close functional relationship between fine‐grained sediment and phosphorus, efficacy ranges for reductions in particulate phosphorus loads can be similar. Our new results for reductions in sediment loss are reasonably well aligned with, although slightly lower, than existing understanding of reductions in sediment loads. Here, it is important to acknowledge that our study represents the establishment phase of the vegetation treatments. On that basis, the overall efficacies for the study period should be viewed as being underestimates of longer‐term performance since treatment effects could be expected to increase as the buffers continue to mature. Previous work has underscored the potential for reductions in sediment loss to be strongly influenced by deposition of incoming sediment along the upslope leading edge of buffer strips due to the initial reduction in runoff velocity and sediment transport capacity (Ligdi & Morgan, 1995; Pearce et al., 1997). Such edge effects were not observed during our experiment.

Excess sediment loss from agricultural land remains a global issue despite the uptake of best management practices. For England and Wales, for example, such elevated sediment losses due to current structural land cover have been estimated to result in £523 M of environmental damage costs annually, with the uptake of best management practices on farms only reducing those societal costs to £462 M (Collins & Zhang, 2016). Buffer strips continue to feature in the mix of best management practices implemented on farms to protect water quality and their uptake by farmers can be facilitated by robust evidence on the efficacy for reducing water pollution. Agricultural runoff encountering a buffer strip meets a more porous and rougher surface, resulting in a reduction in runoff velocity and sediment transport capacity. Here, the vegetation cover generates increased resistance to runoff and sediment transport and the root systems increase the permeability of the soil surface, thereby encouraging infiltration and deposition (Magette et al., 1989; Rose et al., 2003).

Buffer strips can also assist in the management of the sediment problem by stabilizing and reducing the erosion of riverbanks (Bowie, 1995; Kemper et al., 1992) and by displacing sediment generating land management away from watercourses (Wenger, 1999). The beneficial effects of displacement are often, however, less pronounced on heavy meandering watercourses where channel migration drives bank erosion (Williamson et al., 1992). In England Wales, eroding channel banks have been estimated to contribute 22% of the total fine‐grained sediment load delivered rivers and streams (Zhang et al., 2014). The potential beneficial impacts of buffer strips on reducing bank erosion across England and Wales, as well as sediment loss from utilized agricultural land, should therefore be borne in mind given the important role of bank erosion in the excess sediment problem nationally.

When interpreting evidence for buffer strip impacts on sediment loss, it is important to acknowledge various issues which can confound efficacy. Buffer strips can be prone to silting up, especially when soils are saturated (Barfield et al., 1979; Hayes et al., 1979). Under such conditions, deposited sediment is likely to remain unconsolidated and prone to remobilisation, especially when a sequence of extreme storm events occurs or buffer strips are breached by concentrated runoff in preferential flow paths. Sediment trapping by buffer strips is commonly particle size selective with coarser particles preferentially retained (Hayes et al., 1996; Robinson et al., 1996; Hickey & Doran, 2004). Here, particle size selectivity is often buffer width dependent, with narrow 1 m buffer strips only trapping the coarsest particles (Hayes et al., 1979). Vegetation management can influence buffer strip efficacy for reducing incoming sediment loads since, for example, long grass is more prone to lodging, which can permit preferential flow routes and reduced efficacy. Incoming flow mechanisms can influence efficacy for reducing sediment loads with, for example, concentrated flows reducing efficacy (Blanco‐Canqui et al., 2004; Dillaha et al., 1986; Dosskey et al., 2002). At our experimental site, however, pervasive raindrop‐impacted saturation‐excess overland flow has been identified as a primary mechanism for sediment mobilization and delivery, rather than concentrated runoff (Pulley & Collins, 2019). Finally, in real‐world settings, buffer strips serving agricultural land can be bypassed by field drains (Haycock & Muscutt, 1995; McKergow et al., 2003), meaning that the reductions in sediment loads relate to the surface runoff pathway. In England and Wales, a considerable proportion of farmed land has assisted underdrainage in support of productive agriculture (Robinson & Armstrong, 1988), and field drains represent an important sediment delivery pathway (Deasy et al., 2009; Zhang et al., 2016). If assisted underdrainage exists, this means that more engineered buffer strip solutions will be required to deliver multi‐pathway control of sediment pollution in many parts of England and Wales. Such solutions might, for instance, include the cutting back of field drains to permit the construction of artificial wetlands (Lenhart et al., 2016) thereby delivering a “treatment‐train” strategy combining buffer strips and wetlands. Where woody vegetation on buffer strips is harvested, the timing of such management activities will be critical to minimize compaction issues since these could reduce sediment trapping efficacy.

## 5 CONCLUSIONS

Our results herein clearly indicate that the initiation of different buffer strip vegetation treatments can disturb soils and negate sediment trapping efficacy initially which should be borne in mind, especially when communicating early impacts to land managers. Thereafter, the grass treatment matured faster than the willow and deciduous woodland treatments for reducing sediment loss. Regardless of this timeline, all three vegetation treatments delivered some capacity for reducing sediment loss and our results provide new evidence for farmers, catchment managers and policy teams. Clearly, our results in this paper only report reductions in sediment loss delivered by the different buffer strip treatments, but positive impacts on additional priority pollutants for the agricultural sector, including nutrients and pesticides are likely.

## Data Availability

Research data are not shared.
